# Parsing the IL-37-Mediated Suppression of Inflammasome Function

**DOI:** 10.3390/cells9010178

**Published:** 2020-01-10

**Authors:** Ina Rudloff, Holly K. Ung, Jennifer K. Dowling, Ashley Mansell, Laura D’Andrea, Andrew M. Ellisdon, James C. Whisstock, Philip J. Berger, Claudia A. Nold-Petry, Marcel F. Nold

**Affiliations:** 1 Department of Paediatrics, Monash University, Clayton, Victoria 3168, Australia; ina.rudloff@hudson.org.au (I.R.); holly.ung@monash.edu (H.K.U.); philip.berger@hudson.org.au (P.J.B.); claudia.nold@hudson.org.au (C.A.N.-P.); 2 Ritchie Centre, Hudson Institute of Medical Research, Clayton, Victoria 3168, Australia; 3 School of Pharmacy and Biomolecular Sciences, Royal College of Surgeons in Ireland, 123 St Stephens Green, Dublin 2, Ireland; jenniferdowling@rcsi.ie; 4 Centre for Innate Immunity and Infectious Diseases, Hudson Institute of Medical Research, Clayton, Victoria 3168, Australia; ashley.mansell@hudson.org.au; 5 Department of Molecular and Translational Sciences, Monash University, Clayton, Victoria 3168, Australia; 6 Biomedicine Discovery Institute, Monash University, Clayton, Victoria 3168, Australia; laura.dandrea@monash.edu (L.D.); andrew.ellisdon@monash.edu (A.M.E.); james.whisstock@monash.edu (J.C.W.); 7 Australian Research Council Centre of Excellence in Advanced Molecular Imaging, Monash University, Clayton, Victoria 3168, Australia; 8 Monash Newborn, Monash Children’s Hospital, Clayton, Victoria 3168, Australia

**Keywords:** interleukin 37, interleukin 1β, interleukin 18, inflammasome, ASC, caspase-1, pyroptosis, NLRP3, AIM2

## Abstract

Interleukin (IL)-37 is a member of the IL-1 family of cytokines. Although its broad anti-inflammatory properties are well described, the effects of IL-37 on inflammasome function remain poorly understood. Performing gene expression analyses, ASC oligomerization/speck assays and caspase-1 assays in bone marrow-derived macrophages (BMDM), and employing an in vivo endotoxemia model, we studied how IL-37 affects the expression and maturation of IL-1β and IL-18, inflammasome activation, and pyroptosis in detail. IL-37 inhibited IL-1β production by NLRP3 and AIM2 inflammasomes, and IL-18 production by the NLRP3 inflammasome. This inhibition was partially attributable to effects on gene expression: whereas IL-37 did not affect lipopolysaccharide (LPS)-induced mRNA expression of *Il18* or inflammasome components, IL-37-transgenic BMDM displayed an up to 83% inhibition of baseline and LPS-stimulated *Il1b* compared to their wild-type counterparts. Importantly, we observed that IL-37 suppresses nigericin- and silica-induced ASC oligomerization/speck formation (a step in inflammasome activation and subsequent caspase-1 activation), and pyroptosis (−50%). In mice subjected to endotoxemia, IL-37 inhibited plasma IL-1β (−78% compared to wild-type animals) and IL-18 (−61%). Thus, our study adds suppression of inflammasome activity to the portfolio of anti-inflammatory pathways employed by IL-37, highlighting this cytokine as a potential tool for treating inflammasome-driven diseases.

## 1. Introduction

Interleukin (IL)-1β belongs to the IL-1 family, which further comprises the pro-inflammatory cytokines IL-1α, IL-18, IL-33, IL-36α, IL-36β, and IL-36γ; and the anti-inflammatory family members IL-1 receptor antagonist (IL-1Ra), IL-36Ra, IL-37, and IL-38 [1,2,3]. IL-1β is a powerful inducer of inflammation [4,5]; hence, its function must be tightly regulated. Therefore, production and release of IL-1β involves a two-step process. Therein, an inflammatory signal 1 initiates transcription and production of precursor (pro)-IL-1β and pro-IL-18. Examples for signal 1 events include cytokine signaling (for example, triggered by tumor necrosis factor (TNF)) [6], and activation of innate pattern-recognition receptors (PRR) by pathogen-associated molecular patterns, such as lipopolysaccharide (LPS) [7]. A danger signal (signal 2) subsequently activates NOD (nucleotide-binding oligomerization domain)-like receptors (including NLRP1, NLRP3 NLRP6, and NLRC4) or PYHIN (pyrin and HIN)-domain family members (including AIM2 and IFI16). Together, signals 1 and 2 induce formation and activation of a multi-protein complex termed the inflammasome [7]. The various inflammasomes are usually named after the NOD-like receptor or PYHIN protein involved, for example NLRP3 inflammasome, AIM2 inflammasome, etc., and which of these inflammasomes is preferentially activated depends on the type of danger signal. For example, NLRP3 is activated by crystals or microbial toxins, whereas AIM2 recognizes double-stranded DNA [8]. Importantly, inflammasome activity can be regulated by signal 1. For example, signal 1 induces de novo synthesis of inflammasome components such as NLRP3 [7,9]; furthermore, signal 1-induced deubiquitylation, desumoylation, or phosphorylation of NLRP3 can promote inflammasome activity. However, depending on the phospho-site, phosphorylation of NLRP3 can also inhibit inflammasome activation [10]. NLRP3 and AIM2 inflammasomes contain the adaptor protein ASC (apoptosis-associated speck-like protein containing a CARD), whose activity can also be induced or inhibited by signal 1-mediated post-translational modification such as phosphorylation and ubiquitylation [11]. Following inflammasome activation, ASC forms large protein specks by multimerization and recruits the enzyme caspase-1 to the inflammasome. Upon recruitment, caspase-1 self-activates and subsequently cleaves the precursor form of pro-IL-1β and pro-IL-18, thereby generating the mature forms of both pro-inflammatory cytokines [7,12]. Of note, IL-1β can also be processed independent of caspase-1; for example, caspase-8 has been shown to contribute to the maturation of IL-1β [13]. Independently of the maturation of IL-1β and IL-18, inflammasomes trigger pyroptosis, a type of inflammatory cell death that releases danger signals and perpetuates inflammation [14]. Pyroptosis is facilitated by the formation of pores in the cell membrane by gasdermin D following its cleavage by caspase-1 or caspase-11 [15,16].

Failure to control IL-1β production is known to contribute to many debilitating diseases, such as rheumatoid arthritis, urate crystal arthritis (gout), type 2 diabetes [17], and bronchopulmonary dysplasia [18,19]. Therefore, it is not surprising that in addition to the control mechanisms just outlined, various anti-inflammatory mediators contribute to dampening IL-1β bioactivities; among them are members of the IL-1 cytokine family itself, such as IL-37. IL-37 was identified in 2000 by several groups [20,21,22] and is a broadly-acting, potent inhibitor of inflammation induced by the innate and the adaptive arm of the immune system [23,24,25,26]. Of the five IL-37 isoforms, termed IL-37a–e, isoforms a, b, and d can form the β-trefoil structure characteristic of the IL-1 superfamily [25,26,27,28,29]. Among human peripheral blood mononuclear cells, IL-37 is predominantly produced by monocytes, which release it as an alarmin upon activation, whereas myeloid dendritic cells constitutively release the cytokine, possibly to maintain an anti-inflammatory milieu at steady-state [30]. Similar to IL-1β and IL-18, IL-37 exists as a precursor protein that contains a caspase-1 cleavage site [31], and it has been reported that caspase-1 inhibition reduces the maturation and function of IL-37 in macrophages [32,33]. However, IL-37 can also be secreted in the absence of caspase-1 activation [30,32], indicating that other proteases contribute to IL-37 secretion, and/or that it may be functional without cleavage. Intracellular IL-37 can translocate into the nucleus [32] and inhibit inflammation in a Smad3-dependent manner [25]. In addition, extracellular IL-37 binds to its receptor complex consisting of IL-18Rα and IL-1R8 (formerly known as SIGIRR [23]), subsequently activating a multitude of anti-inflammatory pathways [26].

One of the pathways IL-37 uses to mediate its anti-inflammatory program involves the inhibition of the inflammasome [34], a function we explored in detail in this study. We investigated which inflammasomes are targeted by IL-37 and which steps of IL-1β (and IL-18) production and inflammasome activation are affected by IL-37. The murine homolog for IL-37 is yet to be identified, but human IL-37 is functional in mice [35,36,37]. Thus, experiments were carried out in primary and immortalized bone marrow-derived macrophages from a well-established mouse strain transgenic for human IL-37 (IL-37tg, [25]). Overall, the data presented here add knowledge to pathways by which IL-37 bridles inflammation.

## 2. Materials and Methods

### 2.1. Ethics Statement

All experimental procedures performed on animals conformed with the guidelines established by the National Health and Medical Research Council, had the approval of Monash University’s Ethics Committee (MMCA/2012/61 and MMCA/2016/67), and complied with the Declaration of Helsinki.

### 2.2. Animal Experiments

C57Bl/6J wild-type (WT) mice were purchased from Jackson Laboratory (Bar Harbor, ME, USA); mice transgenic for IL-37b (IL-37tg, on C57Bl/6 background) were generated as described previously [25]. IL-37tg animals were back-crossed to WT animals for 10 generations to eliminate potential genetic differences (other than the *Il37* transgene).

A total of 21 WT animals and 20 IL-37tg animals underwent experimentation. Mice received intraperitoneal injections of either lipopolysaccharide (LPS, O55:B5, 10 mg/kg Sigma-Aldrich, St. Louis, MO, USA) or vehicle (saline for injections). Animals had unrestricted access to food and water; room temperature (22 °C) and humidity (50%–60%) were kept constant; and light was cycled in a 12 h day/night rhythm. Twenty-four hours after injection, mice were anaesthetized, and blood was obtained by orbital bleeding into heparinized tubes before the animals were humanely killed. Blood samples were spun (10 min, 300× *g*, 4 °C) and the plasma collected. Plasma samples were snap-frozen in liquid nitrogen and stored at −80 °C until further analysis.

### 2.3. Generation of Bone Marrow-Derived Macrophages (BMDM)

For the generation of primary BMDM (pBMDM), bone marrow was obtained from the femurs and tibiae of humanely killed C57Bl/6 WT or IL-37tg mice. After homogenization of cells by pipetting, erythrocytes were lysed with 1 × BD Pharm Lyse (BD Biosciences, San Diego, CA, USA) for 5 min at room temperature (RT). Afterwards, cells were washed once with DMEM media (Thermo Fisher Scientific, Foster City, CA, USA) and resuspended in DMEM media supplemented with 10% heat-inactivated fetal calf serum (FCS, Thermo Fisher Scientific), 1:500 MycoZap Plus-PR (Lonza, Basel, Switzerland), and 100 U/mL murine M-CSF (Peprotech, Rocky Hill, NJ, USA). Cells were cultured for 6 days at 37 °C and 5% CO_2_ before they were seeded into tissue culture plates (Corning, New York, NY, USA) for subsequent experiments.

To generate immortalized BMDM (iBMDM) using a published J2 recombinant retrovirus carrying v-myc and v-raf/mil oncogenes [38] and a previously described protocol [39], M-CSF-containing conditioned media (L-929 media) and Cre-J2 retrovirus-containing media (Cre-J2 media) were produced: Briefly, L-929 media was produced by growing L-929 cells (NCTC clone 929, ATCC, Manassas, VA, USA) in DMEM supplemented with 10% FCS (Thermo Fisher Scientific) and 1:500 MycoZap Plus-CL (Lonza) at 37 °C with 5% CO_2_. Once 50% confluence was reached, media was replaced (DMEM + 10% FCS without antibiotics) and L-929 cells cultured for another 10 days before conditioned media was retrieved, sterile-filtered, and stored at −80 °C until further use. For Cre-J2 media, Cre-J2 cells were grown in DMEM supplemented with 10% FCS (Thermo Fisher Scientific) and 1:500 MycoZap Plus-CL (Lonza) in a 175 cm^2^ tissue culture flask (Corning) at 37 °C and 5% CO_2_. Once 100% confluent, 75% of cells were passed into a new 175 cm^2^ tissue culture flask and cultured for 24 h before Cre-J2 containing supernatants were harvested, sterile-filtered, and stored at −80 °C until further use. WT and IL-37tg BMDM were differentiated and immortalized simultaneously as follows: Freshly isolated bone marrow cells were seeded in complete media (DMEM supplemented with 10% FCS (Thermo Fisher Scientific), 1:500 MycoZap Plus-PR (Lonza), and 20% L-929 media). After three days of culture at 37 °C and 5% CO_2_, cells were infected by replacing the complete media with fresh complete media supplemented with 50% Cre-J2 supernatants for 24 h before media was changed to complete media without Cre-J2. Another 24 h later, the infection cycle was repeated. After the second infection cycle, cells were cultured in complete media without virus for 7 d before fresh complete media supplemented with only 10% L-929 media was added. Over the next two months, cells were cultured with gradually decreasing concentrations of L-292 media until cells were completely weaned off and survived in the absence of L-929 media. At this stage, proliferating cells were considered immortalized and were either frozen in liquid nitrogen or seeded into tissue culture plates (Corning) for subsequent experiments.

### 2.4. Quantification of Cytokine Concentrations

Plasma concentrations of IL-1β (BD Biosciences) and IL-18 (MBL International, Woburn, MA, USA) were quantified by ELISA according to the manufacturers’ instructions.

Concentrations of IL-1β in cell culture supernatants were analyzed by AlphaLISA, a bead-based immunoassay, according to the protocol provided by the manufacturer (PerkinElmer, Waltham, MA, USA). IL-18 abundance in cell culture supernatants was determined by ELISA (MBL International) as per the manufacturer’s instructions. Where indicated, cytokine abundance in cell culture supernatants was normalized to total protein concentration (determined by BCA assay; Thermo Fisher Scientific).

### 2.5. Inflammasome Activation

For inflammasome activation experiments, 4 × 10^4^ cells were seeded into 96-well tissue culture plates (Corning). Twenty-four hours after seeding, cells were primed with LPS (O55:B5, Sigma-Aldrich) for 3 h, and afterwards, stimulated with nigericin (activation of NLRP3 inflammasome, InvivoGen, San Diego, CA, USA) for 3 h. To activate the AIM2 inflammasome, cells were primed with LPS for 3 h, before being transfected with poly(dA:dT) (InvivoGen) using Lipofectamine 3000 (Thermo Fisher Scientific) according to the manufacturer’s instructions for 6 h. For control purposes, cells were treated with vehicle.

For gene expression experiments, 2 × 10^5^ cells were seeded into 24-well tissue culture plates (Corning). Twenty-four hours later, cells were either treated with vehicle or stimulated with LPS (O55:B5, Sigma-Aldrich) for 3 h.

### 2.6. RNA Isolation and Detection of Gene Expression

Total RNA was isolated with the RNA Mini Kit (Bioline, Sydney, NSW, Australia) following the manufacturer’s instructions, and quantified using a NanoDrop ND-1000 spectrophotometer (Thermo Fisher Scientific). Reverse transcription was performed using the Tetro cDNA Synthesis Kit (Bioline). TaqMan real-time PCRs (TaqMan Gene Expression Master Mix and TaqMan Gene Expression Assay, Thermo Fisher Scientific) were performed according the manufacturer’s protocol on the QuantStudio 6 RT-PCR System (Thermo Fisher Scientific). Samples and no-template controls were run in duplicate for each gene master mix. The following cycling conditions were used: initial denaturation at 95 °C for 10 min, followed by 40 cycles of denaturation at 95 °C for 15 s and annealing/elongation at 60 °C for 1 min. Relative expression quantification was calculated according to Pfaffl’s method [40]. The fold-changes for all gene expression calculations were normalized to 18S ribosomal RNA.

### 2.7. Caspase-1 Activation Assay

Cells were seeded into 12-well tissue culture plates (Corning). After 24 h, cells were stimulated with LPS (O55:B5, Sigma-Aldrich) for 6 h; during the last 2 h of LPS stimulation, nigericin (InvivoGen) was added. Total protein lysates were prepared as previously described [41]; 4 × Laemmli sample buffer (Bio-Rad Laboratories, Hercules, CA, USA) with 10% 2-mercaptoethanol (Sigma-Aldrich) was added, and samples were frozen at −20 °C for subsequent immunoblot analysis. To extract proteins from cell supernatants, protein-binding StrataClean resin (Agilent, Santa Clara, CA, USA) was added; the mixture was vortexed for 30 s and subsequently spun (5 min, 250× *g*, 4 °C). StrataClean resin pellets were resuspended in 4× Laemmli sample buffer (Bio-Rad Laboratories) with 10% 2-mercaptoethanol (Sigma-Aldrich), and stored at −20 °C until being subjected to immunoblot analysis.

### 2.8. ASC Oligomerization Assay

Cells were seeded into 6-well culture plates (Corning). After 24 h, cells were stimulated with LPS (O55:B5, Sigma-Aldrich) for 5 h; during the last 2 h of LPS stimulation, nigericin (InvivoGen) was added. Cells were washed with ice-cold 1 × DPBS (Sigma-Aldrich) before being scraped off the tissue culture plate in ice-cold lysis buffer (20 mM Hepes-KOH pH 7.5, 150 mM KCl, 1% NP-40, 1 mM PMSF, 1:100 Protease Inhibitor Cocktail, all Sigma-Aldrich) and lysed by shearing ten times through a 21 gauge needle (Sigma-Aldrich). Equal proportions of each sample were resuspended in 4× Laemmli sample buffer (Bio-Rad Laboratories) with 10% 2-mercaptoethanol (Sigma-Aldrich) and frozen at −20 °C for subsequent protein input analysis by immunoblot; the remaining samples were spun (10 min, 350× *g*, 4 °C). Freshly prepared 100 mM DSS in DMSO was equilibrated to RT, and then diluted in 1× DPBS (all Sigma-Aldrich) and added to the sample pellets at a final concentration of 2 mM. Samples were incubated for 30 min at RT under rotation and then spun (10 min, 300× *g*, RT). Afterwards, cross-linked pellets were resuspended in 4 × Laemmli sample buffer (Bio-Rad) with 10% 2-mercaptoethanol (Sigma-Aldrich), heated at 95 °C for 5 min, and stored at −20 °C for subsequent immunoblot analysis.

### 2.9. Immunoblot Analysis

Samples from caspase-1 activation and ASC oligomerization assays were heated at 95 °C for 5 min before they were subjected to immunoblot analysis on 10% or 12% SDS-PAGE gels. The following antibodies were used: caspase-1, mouse monoclonal antibody (clone Casper-1, AG-20B-0042-C100) and ASC, rabbit polyclonal antibody (clone AL177, AG-25B-0006-C100), AdipoGen (Liestal, Switzerland); β-actin, mouse monoclonal antibody (clone C4, sc-47778), Santa Cruz Biotechnology (Dallas, TX, USA).

### 2.10. Production and Purification of Recombinant IL-37b-Y85A (recIL-37)

Codon-optimized IL-37b-Y85A (herein called recIL-37) was cloned into a modified tobacco etch virus (TEV) protease–cleavable version of pGEX-4T-1 (GE Healthcare, Chicago, IL, USA) [29]. Recombinant protein was expressed in BL21-CodonPlus(DE3)-RIL cells (Stratagene, San Diego, CA, USA) by isopropyl-β-d-thiogalactopyranoside induction at 18 °C. Cells expressing glutathione S-transferase (GST)–IL-37b-Y85A were lysed by sonication in 20 mM Tris-HCl (pH 8.0), 500 mM NaCl, 1 mM EDTA, and 3 mM β-mercaptoethanol, with two complete EDTA-free protease inhibitor tablets (Roche, Basel, Switzerland). Cells were clarified by centrifugation, filtered through a 0.45-μm membrane, and bound to glutathione sepharose 4B resin (GE Healthcare) for 1 h at 4 °C. The resin was washed with 200 mL of 20 mM Tris-HCl (pH 8.0), 200 mM NaCl, and 3 mM β-mercaptoethanol. The protein was released from the GST tag by overnight incubation with His-TEV protease at 4 °C. The protein was further purified on a MonoS 5/50 GL column (GE Healthcare) equilibrated in 20 mM Tris-HCl pH 7.3 and 50 mM NaCl, and eluted with a 30 column volume gradient from 20 mM Tris-HCl pH 7.3 and 50 mM NaCl to 20 mM Tris-HCl pH 7.3 and 1 M NaCl. Further purification was performed on a HiLoad Superdex 75 16/60 column (GE Healthcare) pre-equilibrated with DPBS (Thermo Fisher Scientific) and 1 mM DTT. Final endotoxin values were less than 1 endotoxin-unit per mg of recombinant protein.

### 2.11. ASC Speck Formation

NLRP3-deficient immortalized macrophages stably expressing ASC-cerulean and NLRP3-Flag (kindly donated by Eicke Latz, University of Bonn, Bonn, Germany), were incubated with recIL-37 (100 pg/mL in 50 μg/mL BSA/PBS) for 1 h at 37 °C. Cells were then incubated with silica (nano-SiO_2_, 250 µg/mL) for 4 h, or nigericin (6 μM) for 90 min at 37 °C. Samples were fixed for 30 min with 4% *w/v* paraformaldehyde and washed with PBS before being imaged on an FV1200 Olympus microscope (Olympus, Tokyo, Japan). Five fields were imaged for each sample containing greater than 100 cells per field. For the quantification of ASC specks, the imaged fields were analyzed as 3-dimensional deconvoluted maximum intensity projections of *z* stacks using an imaging analysis software (ImageJ 2.0.0-rc9/1.49d, Open Source Platform Software).

### 2.12. LDH Assay

Supernatants of cells were analyzed for lactate dehydrogenase (LDH) release as a widely used and accepted indicator for pyroptosis [42,43] according to the instructions of the manufacturer (CytoTox 96 Non-Radioactive Cytotoxicity Assay, Promega, Madison, WI, USA).

### 2.13. Statistical Analysis

Groups were tested for normality and equal variance (*p* to reject 0.05) using GraphPad Prism8 (GraphPad Software, San Diego, CA, USA). Thereafter, one-way ANOVA or ANOVA on ranks was used to test for significant differences between groups. If a significant effect was revealed, post-hoc Sidak or Tukey comparisons were performed (threshold for significance *p* < 0.05). For comparisons between two groups only, a two-tailed Student’s t test was performed.

## 3. Results

### 3.1. IL-37 Inhibits Inflammasome-Mediated Production of IL-1β and IL-18

**Figure 1:**
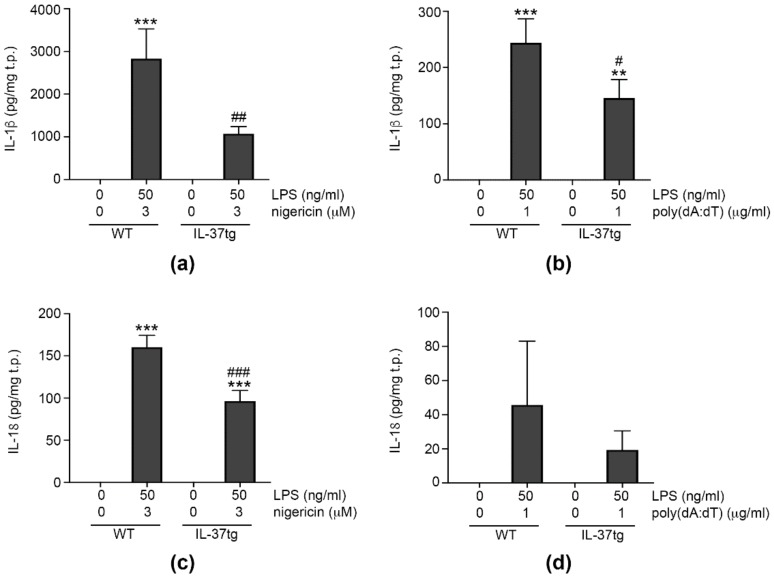
IL-37 inhibits inflammasome-mediated production of IL-1β and IL-18 (**a**–**d**). WT or IL-37tg macrophages were treated with vehicle or primed with lipopolysaccharide (LPS, 50 ng/mL) for 3 h. Cells were subsequently stimulated with 3 μM nigericin for 3 h ((**a**,**c**) *n* = 5 each) or transfected with 1 μg/mL poly(dA:dT) for 6 h (**b**,**d**) (*n =* 7 for (c), *n =* 4 for (d)). IL-1β (**a**,**b**) and IL-18 abundance (**c**,**d**) in cell supernatants were analyzed and cellular total protein content quantified by BCA assay. Graphs show means of cytokine abundance normalized to total protein (t.p.) ± SEMs. **, *p* < 0.01 and ***, *p* < 0.001 for stimulated versus vehicle-treated cells of the same strain; #, *p* < 0.05, ##, *p* < 0.01, and ###, *p* < 0.001 for IL-37tg versus WT macrophages of same treatment. LPS: lipopolysaccharide, tg: transgenic, WT: wild-type.

IL-1β and IL-18 can be produced by different inflammasomes [7,12], and we decided to investigate the effect of IL-37 on IL-1β and IL-18 production by the NLRP3 and/or AIM2 inflammasomes. To study endogenous IL-37 (thus assessing both its intra and extracellular effects [25,26]), we turned to mice transgenic for human IL-37 (IL-37tg) [25]. For inflammasome activation, we primed immortalized bone marrow-derived macrophages (iBMDM) from WT mice or IL-37tg mice with LPS, before providing a second, inflammasome-specific stimulus. As shown in Figure 1a, activation of the NLRP3 inflammasome with the well-characterized NLRP3 agonist nigericin [44] induced robust production of IL-1β in WT cells, whereas there was less IL-1β in IL-37tg macrophages. The difference in IL-1β between WT and IL-37tg macrophages was less pronounced, but still significant when the AIM2 inflammasome was activated with poly(dA:dT) (Figure 1b). Investigating IL-18, we found IL-37tg macrophages produced significantly less cytokine than their WT counterparts upon NLRP3 activation (Figure 1c). AIM2 activation only moderately increased IL-18 in both WT and IL-37tg macrophages; however, this increase was less pronounced in IL-37tg macrophages (difference not statistically significant, Figure 1d).

These findings demonstrate that IL-37 inhibits the production of IL-1β by NLRP3 and AIM2 inflammasomes, and the NLRP3-mediated production of IL-18.

### 3.2. IL-37 Inhibits the mRNA Expression of Il1b

The production of IL-1β and IL-18 is tightly regulated and requires not only the formation and activation of inflammasome complexes (2), but also de novo synthesis of IL-1β and inflammasome components, such as NLRP3 (induced by signal 1) [7,12]. To determine if the observed IL-37-mediated inhibition of IL-1β and IL-18 is due to effects on signal 1, we analyzed the LPS-induced expression of *Il1b* and *Il18* in iBMDM from WT and IL-37tg mice 3 h after stimulation. Moreover, we investigated the effect of IL-37 on the expression of genes encoding proteins with a role in inflammasome-mediated IL-1β maturation, such as NLRP1, NLRP3, NLRC4, and AIM2 (gene names *Nlrp1a*, *Nlrp1b*, *Nlrp3*, *Nlrc4*, and *Aim2*), ASC (*Pycard*), and caspase-1 (*Casp1*). Furthermore, we examined the expression of caspase-8 (*Casp8*), which can cleave pro-IL-1β independently of inflammasome activation [13].

**Figure 2:**
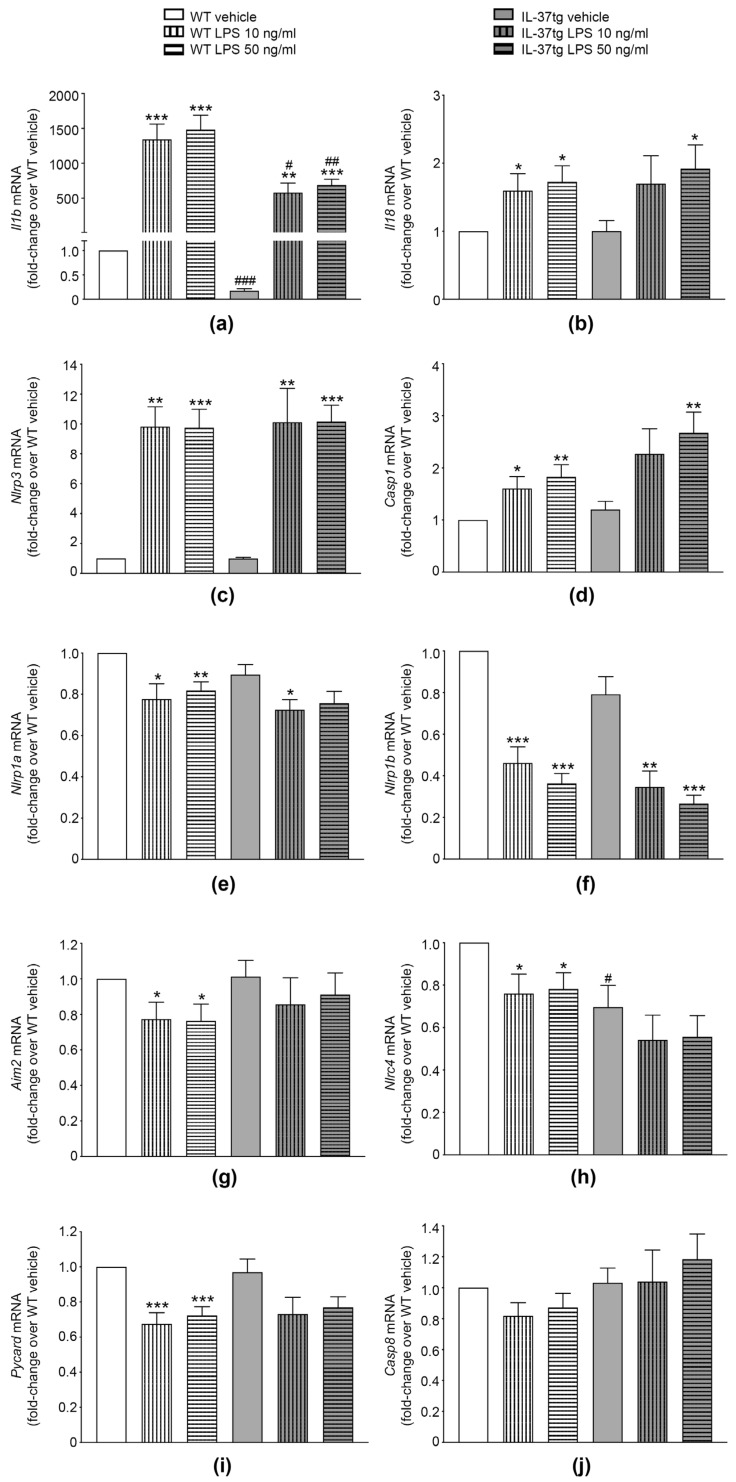
The effects of IL-37 on mRNA expression of *Il1b* and inflammasome components in macrophages. (**a**–**j**) WT or IL-37tg macrophages were treated with vehicle or stimulated with LPS (10 ng/mL or 50 ng/mL) as indicated. After 3 h, total RNA was isolated; gene was expression analyzed by real-time PCR and normalized to the abundance of 18S rRNA. Gene expression is shown as fold-change relative to WT macrophages treated with vehicle (set as 1) ± SEM, *n =* 5–6. *, *p* < 0.05, **, *p* < 0.01, and ***, *p* < 0.001 for LPS versus vehicle-treated cells of the same strain; #, *p* < 0.05, ##, *p* < 0.01, and ###, *p* < 0.001 for IL-37tg versus WT macrophages of same treatment group. LPS: lipopolysaccharide, tg: transgenic, WT: wild-type.

As shown in Figure 2, LPS stimulation for 3 h markedly increased *Il1b* expression (more than 1000-fold in macrophages from both strains when compared to their vehicle-treated counterparts, Figure 2a). Albeit at lower fold-changes, this was also the case for *Il18* (up to 2-fold in both strains, Figure 2b), *Nlrp3* (up to 10-fold, Figure 2c), and *Casp1* (up to 2-fold, Figure 2d); however, LPS stimulation decreased the expression of *Nlrp1a* (up to −22% in WT and −19% in IL-37tg, Figure 2e) and *Nlrp1b* (up to −64% in WT and −66% in IL-37tg, Figure 2f). Furthermore, in WT macrophages, LPS moderately but significantly reduced the expression of *Aim2* (up to −24%, Figure 2g), *Nlrc4* (up to −24%, Figure 2h) and *Pycard* (up to −33%, Figure 2i), while IL-37tg macrophages only showed a non-significant trend towards reduction in these genes (up to −15%, −22%, and −25%, respectively, Figure 2g–i). The expression of *Casp8* was not significantly affected by LPS stimulation in either WT or IL-37tg macrophages (Figure 2j). We also did not observe any difference in the expression of the genes investigated comparing 10 ng/mL with 50 ng/mL of LPS (Figure 2a–j).

When compared to the LPS-induced gene expression in WT macrophages, however, IL-37tg macrophages showed a marked reduction in the LPS-induced expression of *Il1b* (reduction of up to −57%, Figure 2a). Importantly, IL-37tg cells also expressed markedly less *Il1b* mRNA at steady-state (−83%, Figure 2a). Interestingly, there were no differences in LPS-regulated expressions of *Il18*, *Nlrp3*, *Casp1*, *Nlrp1a*, *Nlrp1b*, *Aim2*, *Nlrc4*, *Pycard*, or *Casp8* between WT and IL-37tg macrophages, highlighting the specificity in *Il1b* regulation by IL-37. We also observed no difference in the expression of aforementioned genes in vehicle-treated WT compared to IL-37tg cells (Figure 2b–j), with the exception of *Nlrc4*, of which IL-37tg cells produced 30% less than WT cells at steady-state (Figure 2h).

In summary, we show that while IL-37 significantly inhibits the expression of *Il1b* both at steady-state and upon LPS stimulation, it does not affect the LPS-mediated expression of *Il18* or any of the inflammasome components investigated here.

### 3.3. IL-37 Inhibits ASC Multimerization

Next, we investigated whether IL-37 inhibits inflammasome activation; i.e., signal 2. ASC homotypic dimerization is required to facilitate formation of several inflammasomes, such as NLRP3 and AIM2, leading to the recruitment and subsequent cleavage and activation of caspase-1, IL-1β and IL-18 [7]. Hence, to determine whether IL-37 suppressed inflammasome activation directly, we examined the effects of IL-37 on ASC multimerization.

**Figure 3:**
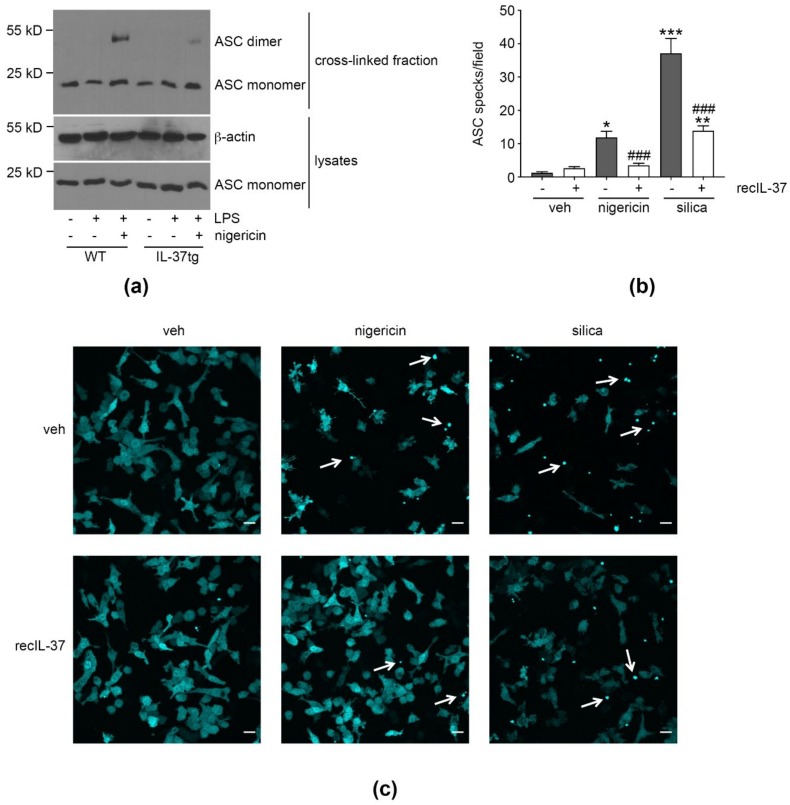
IL-37 inhibits ASC oligomerization. (**a**) WT or IL-37tg macrophages were treated with LPS (500 ng/mL) or vehicle, as indicated. After 3 h, nigericin (10 μM) was added for another 2 h. ASC and β-actin were quantified. Depicted is one representative blot of three independent experiments. (**b**,**c**) ASC-cerulean reporter macrophages were treated with recIL-37 (100 pg/mL) for 1 h, as indicated, before being stimulated with nigericin (6 μM) or silica (250 ng/mL) for 90 min or 4 h, respectively. Control cells were treated with vehicle. Cells were imaged with confocal microscopy and images flattened to allow maximum-intensity projections of 3-dimenisonal deconvolved z-stacks. (**b**) The number of specks per field of view ± SEM is depicted. Results are from two independent experimental replicates, in each of which five fields per sample were imaged, and each field contained >100 cells. *, *p* < 0.05, **, *p* < 0.01, and ***, *p* < 0.001 for nigericin- or silica-treated cells (with or without recIL-37 pre-stimulation as indicated) compared to vehicle-treated cells that were not pre-stimulated with recIL-37. ###, *p* < 0.001 for recIL-37 compared to vehicle within the same stimulation condition (nigericin or silica). (**c**) Depicted are representative images of (**b**). White arrows indicate ASC specks; scale bars 20 μm. LPS: lipopolysaccharide, tg: transgenic, veh: vehicle, WT: wild-type.

Assessing ASC oligomerization by immunoblot, we detected the expected induction of ASC dimerization in iBMDM from WT and IL-37tg mice treated with LPS (signal 1) + nigericin (signal 2), but not in cells deprived of signal 2. Importantly, ASC dimerization was reduced in LPS/nigericin-stimulated IL-37tg macrophages compared to WT cells (Figure 3a).

To confirm this observation in a different experimental setting, we assessed the formation of ASC specks [45] using ASC-cerulean macrophages [46] stimulated with activators of the NLRP3 inflammasome as a readout of inflammasome activation. Importantly, ASC-cerulean macrophages are derived from WT mice, and thus, are devoid of endogenous IL-37. To assess the effect of IL-37 on speck formation, ASC-cerulean cells were, thus, treated with recombinant (rec)IL-37. Whereas untreated macrophages displayed a robust induction of ASC speck formation following nigericin challenge, pre-treatment of macrophages with recIL-37 [29] strongly reduced nigericin-induced speck formation (Figure 3b,c). Similarly, speck formation induced by silica, a crystalline activator of the NLRP3 inflammasome, was lower in cells pre-treated with recIL-37.

Taken together, these findings show that IL-37 potently inhibits the signal 2-induced multimerization of ASC in mouse macrophages.

### 3.4. IL-37 Inhibits Caspase-1 Activation

Upon association with NLPR3 and subsequent multimerization, ASC recruits caspase-1 p45 via homotypic CARD-CARD interaction, leading to autocatalytic cleavage of caspase-1 into its active p10 and p20 subunits. As inflammasome activation also induces macrophage cell death by pyroptosis, pro-caspase-1 and its mature, cleaved caspase-1 fragments are released from cells [47] and can be detected in cell supernatants.

**Figure 4:**
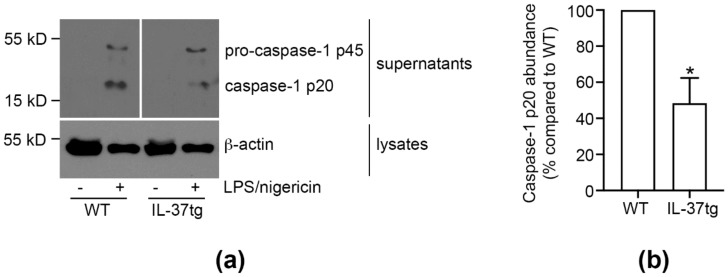
IL-37 inhibits caspase-1 activation. (**a**,**b**) WT or IL-37tg macrophages were stimulated with LPS (50 ng/mL) as indicated. After 4 h, nigericin (3 μM) was added for another 2 h. Control cells were treated with vehicle. Caspase-1 (pro-caspase-1 p45 and cleaved caspase-1 p20) and β-actin were assessed by immunoblot. Depicted is (**a**) one representative blot of three independent experiments (lanes irrelevant to this study were cut from the immunoblot images) and (**b**) the caspase-1 p20 signal intensity quantification normalized to β-actin. Caspase-1 p20 protein abundance is presented as change in caspase-1 p20 compared to WT (set as 100%) ± SEM. *, *p* < 0.05 for IL-37tg compared to WT. LPS: lipopolysaccharide, tg: transgenic, WT: wild-type.

To investigate whether inhibition of ASC multimerization by endogenous IL-37 in macrophages also leads to a subsequent decrease in the activation of caspase-1, we performed caspase-1 activation assays in WT and IL-37tg primary BMDM (pBMDM) stimulated to activate the NLRP3 inflammasome. Whereas the p20 fragment of cleaved caspase-1 was not detectable in supernatants of vehicle-treated WT or IL-37tg macrophages, activation of the NLRP3 inflammasome with LPS and nigericin led to the release of p20 and p45 into cell supernatants (Figure 4a). In IL-37tg macrophages, release of cleaved caspase-1 p20 was reduced by 52% when compared to their WT counterparts (Figure 4b).

Thus, we show that the IL-37-mediated inhibition of ASC multimerization leads to a reduction of caspase-1 activation in murine macrophages.

### 3.5. IL-37 Inhibits Pyroptosis

Inflammasome activation induces pyroptosis, a process mediated by caspase-1 or caspase-11 and gasdermin D [15,16], resulting in cell death and the release of LDH [42,43]. Although IL-37 failed to reduce *Casp1* mRNA expression (Figure 2d), it inhibited the processing of caspase-1 protein (Figure 4a,b). Therefore, we next investigated whether IL-37 also inhibits pyroptosis.

**Figure 5:**
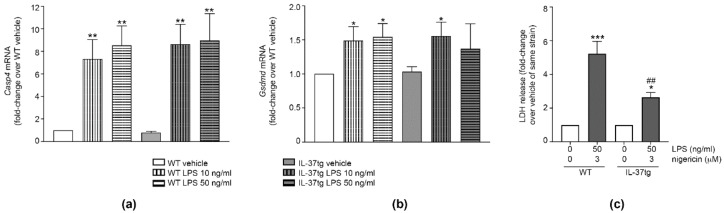
IL-37 inhibits pyroptosis. (**a**,**b**) WT or IL-37tg macrophages were treated with vehicle or stimulated with LPS (10 ng/mL or 50 ng/mL) as indicated. After 3 h, total RNA was isolated; gene expression was analyzed by real-time PCR and normalized to the abundance of 18S rRNA. Gene expression of *Casp4* (**a**) or *Gsdmd* (**b**) is shown as fold-change relative to WT macrophages treated with vehicle for the same duration (set as 1) ± SEM; *n =* 6. *, *p* < 0.05 and **, *p* < 0.01 for LPS versus vehicle-treated cells of the same strain. (**c**) WT or IL-37tg macrophages were stimulated with LPS as indicated. After 3 h, nigericin was added for another 3 h. Control cells were treated with vehicle. LDH was measured in cell supernatants and LDH release is depicted as fold-change relative to vehicle-treated cells of the same strain (set as 1) ± SEM; *n =* 5. *, *p* < 0.05 and ***, *p* < 0.001 for LPS/nigericin versus vehicle-treated cells of the same strain; ##, *p* < 0.01 for IL-37tg versus WT macrophages of same treatment group. LPS: lipopolysaccharide, tg: transgenic, WT: wild-type, LDH: lactate dehydrogenase.

While treatment of iBMDM with different concentrations of LPS increased the expression of the mRNA encoding both caspase-11 (gene name *Casp4*, Figure 5a) and gasdermin D (*Gsdmd*, Figure 5b), we did not observe differences in these genes between WT and IL-37tg macrophages (Figure 5a,b). Stimulation with LPS/nigericin increased LDH release by both WT and IL-37tg macrophages. However, LPS/nigericin-stimulated IL-37tg macrophages released 50% less LDH than WT macrophages (Figure 5c). Thus, IL-37 inhibits pyroptosis independently of *Casp4* or *Gsdmd* mRNA expression.

### 3.6. IL-37 Inhibits the LPS-Induced Production of IL-1β and IL-18 In Vivo

Having demonstrated an inhibitory effect of IL-37 on the mRNA expression of *Il1b*, ASC oligomerization, and the activation of caspase-1 in vitro, we next explored the production of IL-1β in vivo in a mouse model of endotoxic shock [25,26,29].

**Figure 6:**
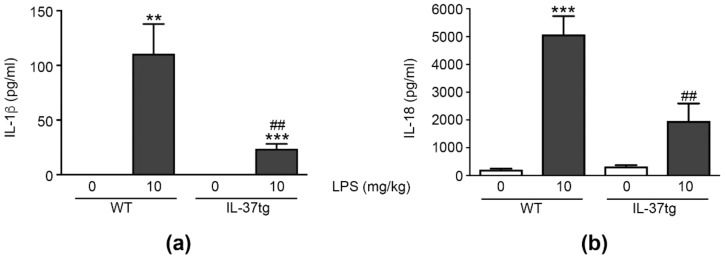
IL-37 inhibits the LPS-induced increase in plasma IL-1β and IL-18 in vivo. (**a**,**b**) WT or IL-37tg mice were injected intraperitoneally with LPS (10 mg/kg) or vehicle. After 24 h, plasma abundance of IL-1β (**a**) or IL-18 (**b**) was detected by ELISA. Data are shown as means ± SEMs; *n =* 8–12 animals per group. ** *p* < 0.01 and *** *p* < 0.001 for LPS versus vehicle-injected animals of the same strain; ## *p* < 0.01 for LPS-injected IL-37tg versus LPS-injected WT mice. LPS: lipopolysaccharide, tg: transgenic, WT: wild-type.

While IL-1β was undetectable in the plasma of vehicle-treated mice, we measured substantial amounts of IL-1β in the plasma of LPS-injected WT and IL-37tg animals. However, in comparison to LPS-injected WT mice, IL-37tg animals treated with LPS had 78% less plasma IL-1β (111 ± 27 pg/mL versus 24 ± 5 pg/mL, respectively; Figure 6a).

Like IL-1β, IL-18 undergoes enzymatic maturation by caspase-1 [7,12]. To investigate the effect of IL-37 on the generation of mature IL-18, we also analyzed LPS-induced plasma IL-18 in mice subjected to endotoxemia (Figure 6b). WT mice exhibited a significant increase in IL-18 plasma concentrations (24-fold increase over vehicle-injected animals) following LPS challenge, while IL-37tg animals demonstrated a 61% reduction in LPS-induced plasma IL-18 compared to WT mice (5077 ± 661 pg/mL versus 1963 ± 632 pg/mL, respectively).

These data show that endogenous IL-37 markedly reduces the pro-inflammatory cytokines IL-1β and IL-18 in the plasma of mice with endotoxic shock.

## 4. Discussion

Over the last decade, IL-37 has emerged as a powerful and broad inhibitor of inflammation [23,24,25], but much remains to be learned regarding the mechanisms employed by IL-37 to facilitate its anti-inflammatory program. Our study identifies a dual role for IL-37 in suppressing IL-1β-mediated inflammation: we show that IL-37 inhibits *Il1b* expression by activated macrophages (signal 1), and importantly reveal that IL-37 inhibits the ASC oligomerization that precedes caspase-1 activation (signal 2) of inflammasome function. Thereby, our study significantly enhances knowledge on the anti-inflammatory effects IL-37 exerts on the inflammasome-mediated biological maturation of IL-1β. Furthermore, we show that, despite potently inhibiting IL-18 production, IL-37 does not affect *Il18* expression in activated macrophages; thus, revealing marked differences in the IL-37-mediated inhibition of IL-1β and IL-18.

Investigating the effect of IL-37 on the LPS-mediated expression of *Il1b*, *Il18*, or inflammasome components, we found that IL-37 inhibits the LPS-mediated induction of IL-1β production. This finding is consistent with the IL-37-mediated inhibition of *Il1b* expression reported in lung lysates of Aspergillus fumigatus-infected mice [34]. That same study also found IL-37-mediated inhibition of *Nlrp3* mRNA [34], which we did not observe here. This discrepancy is likely due to differences in the experimental setting; i.e., total lung tissue versus macrophages, and in vivo aspergillosis versus in vitro LPS stimulation. Besides the absence of an effect of IL-37 on *Nlrp3* expression, we also found no effect of IL-37 on the steady-state or LPS-mediated expression of *Il18*, *Casp1*, *Nlrp1a*, *Nlrp1b*, *Aim2*, *Pycard*, and *Casp8*, while for *Nlrc4* we observed a moderate, but significant reduction in IL-37tg macrophages at steady-state but not upon LPS stimulation. These results indicate that the IL-37-mediated inhibition of LPS-induced IL-1β production by different inflammasomes is attributable to a reduction in *Il1b* mRNA, but not to a decrease in mRNA expression of inflammasome components. In this context, it is noteworthy that LPS-activated macrophages switch their immunometabolism from oxidative phosphorylation to aerobic glycolysis and accumulate succinate. Succinate stabilizes hypoxia-inducible factor 1α (HIF-1α), which in turn induces *Il1b* [48]. Whether IL-37 specifically inhibits *Il1b* mRNA by targeting the succinate/HIF-1α/IL-1β-axis or by inhibiting glycolysis in activated macrophages would be an interesting topic for future studies. However, the hypothesis that IL-37 inhibits *Il1b* expression by regulating the immunometabolism of activated macrophages is certainly supported by the finding that LPS-stimulated THP-1 cells overexpressing IL-37 display a pseudo-starvational state [26]. This state is characterized by the inhibition of the anabolic mTOR (mammalian target of rapamycin) pathway and the activation of the catabolic AMP-activated kinase, ultimately resulting in anti-inflammation [26].

The LPS-mediated decreases in *Nlrp1a*, *Nlrp1b*, *Aim2*, *Nlrc4*, and *Pycard* mRNA we observed in both WT and IL-37tg macrophages were unexpected. A study conducted in human monocytes and macrophages [49] reported that the expression of inflammasome components depends on pre-treatment of the cells, with expression profiles differing between cells treated to differentiate into what the authors define as M0, M1, and M2 macrophages. Consistent with what we observed here, LPS stimulation substantially decreased the expression of *PYCARD* in non-pre-treated (equivalent to M0 in [49]) macrophages, and the authors also observed a trend towards reduced *NLRC4* in these cells [49].

Mouse macrophages constitutively express *Il18* [50], and, in accordance with published studies [51], we found stimulation with LPS further increased its expression, albeit moderately compared to *Il1b*. However, IL-37 did not affect *Il18* mRNA expression in our study. In light of a strong inhibition of *Il1b* mRNA by IL-37 and the significant attenuation of LPS-mediated maturation of both IL-1β and IL-18 in vitro (through the NLRP3 inflammasome) and in vivo, the absence of an effect of IL-37 on *Il18* expression was somewhat unexpected. A possible explanation for this finding could lie in the fact that while IL-1β is produced by myeloid cells, IL-18 can also be generated by epithelial and endothelial cells [1]. In fact, the absence of an effect of IL-37 on *Il18* mRNA might reflect that IL-37 shifts inflammatory responses away from myeloid cells and IL-1β towards non-myeloid cells and IL-18; a speculation in line with our observation that the IL-37-mediated reduction in IL-18 protein is less pronounced than that in IL-1β. In this context, it is also noteworthy that IL-18 constitutively released by epithelial cells of the intestine is important for tight junction formation, barrier integrity, and gut homeostasis [52]; hence, selective blockade of IL-1β but not IL-18 de novo synthesis may have physiological advantages. Differential regulation at the promoter level might also play a role, as in contrast to IL-1β, IL-18 production does not depend on signal 1 [53,54].

Besides inhibiting signal 1 for IL-1β production, we also show that IL-37 markedly reduces the effects of signal 2 by suppressing ASC multimerization that precedes caspase-1 activation. These results not only further document the breadth of IL-37′s activity in inhibiting IL-1β-induced inflammation by affecting multiple steps required for IL-1β maturation, but also confirm that IL-37 inhibits events that occur early in inflammatory responses (i.e., IL-37 inhibiting signal 1 for *Il1b* expression). This is in line with a previous study, in which we observed that human CD14^+^ monocytes release IL-37 as early as 3 h after LPS stimulation, concluding that IL-37 is an anti-inflammatory alarmin [30].

In this study, we did not further explore the mechanistic underpinnings of the effects described above. In this context it is important to note that inflammasome activity can be controlled by multiple mechanisms: In addition to the signal 1-mediated post-translational regulation of NLRP3 or ASC through phosphorylation, (de)ubiquitylation, and (de)sumoylation, which can activate or inhibit their functions [10,11], inflammasome activity can be modulated by interaction with decoy proteins such as pyrin-only proteins (POPs) or CARD-only proteins (COPs). For example, POPs can inhibit ASC-NLRP3 interactions, and COPs can bind to pro-caspase-1 and prevent its activation [10]. However, it should be noted that both POPs and COPs are found in primates only [10]. It is yet to be determined whether in the mouse macrophages studied herein, IL-37 directly interacts with inflammasome components such as ASC or suppresses post-translational modification of inflammasome components. However, our study highlights that IL-37 employs multiple strategies to suppress the inflammasome-mediated inflammatory bioactivity of IL-1β.

Caspase-1 and caspase-11 (gene name *Casp4*) can activate gasdermin D to induce pyroptosis [15,16], a form of programmed cell death that leads to the release of cytosolic contents of affected cells [55]. As IL-37 inhibits the activation of caspase-1, we subsequently investigated the effect of IL-37 on pyroptosis. While IL-37 did not reduce LPS-induced mRNA expression of *Casp1*, *Casp4*, and *Gsdmd* in murine macrophages, it substantially reduced pyroptosis. Being an inflammatory form of cell death [14,55], pyroptosis has been linked to the clearance of infections [56], but also to disease progression in sepsis, vascular inflammation, and cardiovascular disease [57,58]. Therefore, the inhibition of pyroptosis by IL-37 and the corresponding reduction of the release of inflammatory mediators by affected cells can be regarded as another means by which IL-37 inhibits excessive inflammation.

Excessive or dysregulated inflammasome activation is known to be involved in numerous autoinflammatory and autoimmune diseases, such as CAPS (cryopyrin-associated periodic syndromes), FMF (familial Mediterranean fever), PAPA (pyogenic arthritis, pyoderma gangrenosum and acne), gout, or type II diabetes. While some of these illnesses are caused by mutations in inflammasome components (e.g., CAPS, FMF), others are due to indirect effects on inflammasome activation (e.g., PAPA, gout, type II diabetes) [59,60]. Although these diseases are all characterized by excessive inflammation and are amenable to IL-1β blocking with IL-1 receptor antagonists or neutralizing antibodies [17], it is well known that anti-IL-1β therapy is not effective in all patients or can lose efficacy over time [17,61,62,63]. Therefore, new anti-inflammatory treatment options are highly sought-after. It is tempting to speculate that patients suffering from severe IL-1β- and inflammasome-driven inflammatory diseases would benefit from the development of anti-inflammatory therapies employing IL-37. In fact, IL-37 has been shown to ameliorate inflammation in experimental gout [64] and to be protective against obesity-induced inflammation and insulin resistance that can lead to type II diabetes [35].

In conclusion, our study demonstrates that IL-37 employs multiple strategies to suppress the bioactivity of inflammasomes and consequently of IL-1β and IL-18. In macrophages, IL-37 reduces both IL-1β and IL-18 on the protein level, but only *Il1b* on the mRNA level. IL-37 potently reduces inflammasome activation (as demonstrated by marked reductions in ASC oligomerization and caspase-1 activation) and pyroptosis in vitro. Importantly, in an in vivo model of excessive inflammation, IL-37 also reduces IL-1β and IL-18 plasma concentrations upon LPS challenge. Thus, our study substantially enhances the knowledge of the anti-inflammatory functions of IL-37, and, importantly, makes a compelling case for investigations into IL-37 as a therapeutic for IL-1β and inflammasome-driven inflammation.
